# Influenza A Viruses and Zoonotic Events—Are We Creating Our Own Reservoirs?

**DOI:** 10.3390/v13112250

**Published:** 2021-11-09

**Authors:** Susanne Kessler, Timm C. Harder, Martin Schwemmle, Kevin Ciminski

**Affiliations:** 1 Medical Center, Institute of Virology, University of Freiburg, 79104 Freiburg, Germany; susanne.kessler@uniklinik-freiburg.de (S.K.); martin.schwemmle@uniklinik-freiburg.de (M.S.); 2 Faculty of Medicine, University of Freiburg, 79104 Freiburg, Germany; 3 Friedrich-Loeffler-Institut (FLI), Institute of Diagnostic Virology, 17493 Greifswald-Insel Riems, Germany; Timm.Harder@fli.de

**Keywords:** influenza A viruses, zoonosis, livestock farming, pandemic, animal-human interface, avian influenza, swine influenza, equine influenza

## Abstract

Zoonotic infections of humans with influenza A viruses (IAVs) from animal reservoirs can result in severe disease in individuals and, in rare cases, lead to pandemic outbreaks; this is exemplified by numerous cases of human infection with avian IAVs (AIVs) and the 2009 swine influenza pandemic. In fact, zoonotic transmissions are strongly facilitated by manmade reservoirs that were created through the intensification and industrialization of livestock farming. This can be witnessed by the repeated introduction of IAVs from natural reservoirs of aquatic wild bird metapopulations into swine and poultry, and the accompanied emergence of partially- or fully-adapted human pathogenic viruses. On the other side, human adapted IAV have been (and still are) introduced into livestock by reverse zoonotic transmission. This link to manmade reservoirs was also observed before the 20th century, when horses seemed to have been an important reservoir for IAVs but lost relevance when the populations declined due to increasing industrialization. Therefore, to reduce zoonotic events, it is important to control the spread of IAV within these animal reservoirs, for example with efficient vaccination strategies, but also to critically surveil the different manmade reservoirs to evaluate the emergence of new IAV strains with pandemic potential.

## 1. Introduction

Influenza A viruses (IAVs) are the causative agent for seasonal epidemics in the human population and account for a substantial morbidity and mortality that results in a considerable economic burden [1,2]. The ability of IAVs to rapidly cross interspecies barriers and circulate in a variety of avian and mammalian species of wildlife and livestock creates a breeding ground for zoonotic strains with pandemic potential. Since the beginning of the 20th century, zoonotic spillover events have given rise to the generation of multiple, well-documented pandemic IAVs [3,4,5]. Intensification of animal husbandry, increasing encroachment into wildlife habitats for agricultural use, and increased connectivity of livestock populations through (transboundary) trade, created favorable conditions that are associated with the establishment of new IAV lineages in these reservoirs but also created new interfaces for human infections [6,7,8,9,10,11]. Some examples include the 2009 pandemic swine influenza virus [12] and an increasing number of reported zoonotic spillover infections with avian IAVs (AIVs) from poultry [13,14]. Hence, mechanisms need to be in place to prevent zoonotic transmissions and lurking epidemics or even pandemics. As outlined below in more detail, it is important to understand that humans had significant influence on the generation, broadening, and deepening of some of the IAV reservoirs. We therefore have to be aware of these viral reservoirs and have to monitor emerging and circulating virus strains. In the following, we will introduce human-relevant IAV hosts species, the respective host-human interfaces, and discuss their importance for zoonotic spillover.

## 2. Viral Properties

IAVs are subtyped based on the genetic and antigenic properties of their two major surface glycoproteins, hemagglutinin (HA) and neuraminidase (NA). While HA mediates cell attachment and subsequent host cell entry, NA possesses a receptor-destroying function that facilitates egress from an infected cell [5]. To date, 18 HA (H1–18) and 11 NA (N1–11) subtypes are described [15]. Due to their exposed location on the virion surface, HA and NA are subject to selection pressure by the humoral immunity. In a process that is known as antigenic drift, HA and NA undergo changes by the selection of point mutations that randomly occur during genome amplification and allow immune escape. Antigenic drift is the reason why human IAVs can overcome immunity in the human population and why seasonal adjustments to the associated vaccines are required. Aside from these minor changes, IAVs are also capable of altering their genome composition. The segmented IAV genome favors a process called reassortment, in which genomic information is exchanged upon co-infection of a single cell with at least two distinct IAV strains. This exchange of genome segments enables a rapid viral evolution that can lead to the generation of novel antigenic combinations to which the human population is immunologically naïve (antigenic shift) [16].

## 3. IAV Pandemics in Humans

It was not until 1933 that Smith and colleagues isolated IAV from throat-washing samples of infected human patients [17]. Hence, influenza outbreaks that occurred prior to the first third of the 20th century have been assigned to influenza viruses either by clinical manifestation or retrospectively by the isolation of the virus from preserved tissue. The first influenza pandemic in the late modern period that is thought to be caused by an IAV was the so-called Russian flu in 1889–1890. The Russian flu most likely emerged first in the Central Asian part of the Russian Empire and spread to the west along the trade routes to Europe, the US, and finally to Africa and Asia, and accounted for a significant number of deaths [18]. A series of sero-archaeological studies that were carried out in the 1950s and 1960s suggested that an H2N2 virus caused the 1889–1890 pandemic [19,20], whereas other studies have failed to provide evidence for a pandemic H2N2 virus [21,22,23]. In 1965, serological studies found that antibodies were reactive to horse-derived H3 in the majority of people born before 1891 [24,25,26]. It is thus believed that a horse-derived H3N8 was the causative agent of the Russian flu [27]. Others, however, assume that, based on the clinical picture, the pandemic was caused by a zoonotic coronavirus [28,29]. Since the beginning of the 20th century, four major and well-documented IAV pandemics have occurred. The first and most devastating pandemic with about 50 million deaths was the 1918 Spanish flu that was caused by an H1N1 virus. It is currently unclear whether the 1918 H1N1 virus was spilled-over directly from avian species to humans or whether it was transmitted from an intermediate host [30]. Furthermore, it is unclear where the virus first emerged. One theory is that the pandemic virus emerged in an American military camp in Kansas and from there the Spanish flu reached the battlefields in Western Europe after the US entered World War I. Others suspect that the virus may have emerged at a large military base on the Western Front, where 100,000 soldiers lived in close proximity to several farms with poultry, geese, and swine, and were frequently exposed to various gases of war causing respiratory irritation and distress [31,32,33]. In any case, it is very likely that World War I contributed to the spread of the virus. Following the pandemic outbreak, the 1918 pandemic H1N1 virus became endemic to the human population. In 1957, the Asian flu (H2N2) emerged as a reassortant of an avian H2N2 virus and the seasonal descendant of the 1918 H1N1 strain in China [34,35]. The pandemic virus became endemic thereafter and continued to circulate as a seasonal strain, displacing the previous seasonal H1N1 strain [36,37]. Similarly, the 1968 Hong Kong Influenza was caused by genetic reassortment of the antigenically drifted H2N2 virus and an avian H3N? virus giving rise to the pandemic H3N2 subtype [34,35]. The seasonal descendant of the pandemic H3N2 replaced the human H2N2 strain and continues circulating to date. Intriguingly, in 1977, almost 20 years after its disappearance, a human H1N1 virus re-emerged in northern China causing a minor pandemic outbreak, and has been co-circulating with the H3N2 seasonal strain in the human population ever since [38,39]. As this virus exhibited a striking similarity on nucleotide level to seasonal H1N1 of the 1950s, it is now thought that the re-introduction is due to a manmade laboratory accident or failed vaccine trials [39]. Importantly, the Spanish flu, Asian flu, and the Hong Kong flu either emerged directly from an avian virus or were the result of a reassortment event of a human seasonal strain with an avian virus. In contrast, the most recent 2009 pandemic was caused by a zoonotic transmission of a H1N1 swine influenza virus. The 2009 pandemic virus emerged as a quadruple reassortant of a triple reassortant virus of the North American swine lineage, containing gene segments of avian, swine, and human origin and an H1N1 Eurasian avian-like swine virus from swine [40].

In summary, all of the pandemic IAV strains since 1889 have emerged from an animal host either in whole or in part as a reassortant with a human or mammalian adapted virus.

## 4. Avian Reservoir Species

To date all of the known classical HA (H1–16) and NA (N1–9) subtypes (notable exceptions are H17–18 and N10–11) have been found in wild aquatic birds and were also isolated from many of these species, such as ducks, geese, and gulls, although H13 and H16 seem to be restricted to some species of the order *Charadriiformes*, most prominently gulls [41,42,43,44,45,46,47]. Aquatic wild birds of the order *Anserifomes* and *Charadriiformes* are thus believed to constitute the natural IAV reservoir [5]. However, the migratory behavior of many of these species and their overlapping habitats with other animals has led to a global dissemination, introduction, and adaptation of AIVs into new host populations [48]. Consequently, transmitted IAVs have established several host-specific lineages in humans, swine, and horses.

Based on phylogenetic analysis of the viral nucleoprotein sequence, IAVs have established three lineages in birds, one in gulls and two geographically separate lineages that correspond to the flyways of migratory birds, the Eurasian (including the avian-like swine lineage) and the American lineage [49,50,51]. Infections of aquatic birds with AIVs typically affect the cells lining the intestinal and respiratory tracts and proceed asymptomatically or cause only mild disease. As a result, of the intestinal replication, infectious virus is shed in high concentrations through the feces [52,53], leading to a fecal-oral transmission of the virus via contaminated water or feed. Clinically and genetically, two IAV pathotypes of the H5 and H7 subtypes can be distinguished. Low pathogenicity (LP) AIVs (LPAIVs) represent the natural reservoir-like IAVs, which circulate in aquatic wild birds. If transmitted and adapted to replication in galliform poultry (e.g., chicken, turkey, or quail), LP H5 and H7 viruses have the potential to mutate to a high pathogenicity (HP) phenotype, enabling systemic infection. Thus, infections with HP AIVs (HPAIVs) of the H5 and H7 subtypes can cause severe illness and, depending on the viral strain and host-specific parameters, a high mortality of up to 100% in a flock [5,52,54]. Similar to the course in aquatic wild birds, infections with LPAIVs often cause only mild disease in domestic waterfowl and galliform poultry. LPAI outbreaks in poultry farms were shown to be associated with spatial proximity to waterways and, for free-range poultry, with the proximity to habitats of wild waterfowl [55]. Importantly, while HPAIVs are naturally absent in aquatic wild birds, they can be re-transmitted from galliform poultry to wild birds and vice versa. This aids the distribution of the viruses along the migratory routes of the birds [56,57,58,59,60].

**Figure 1:**
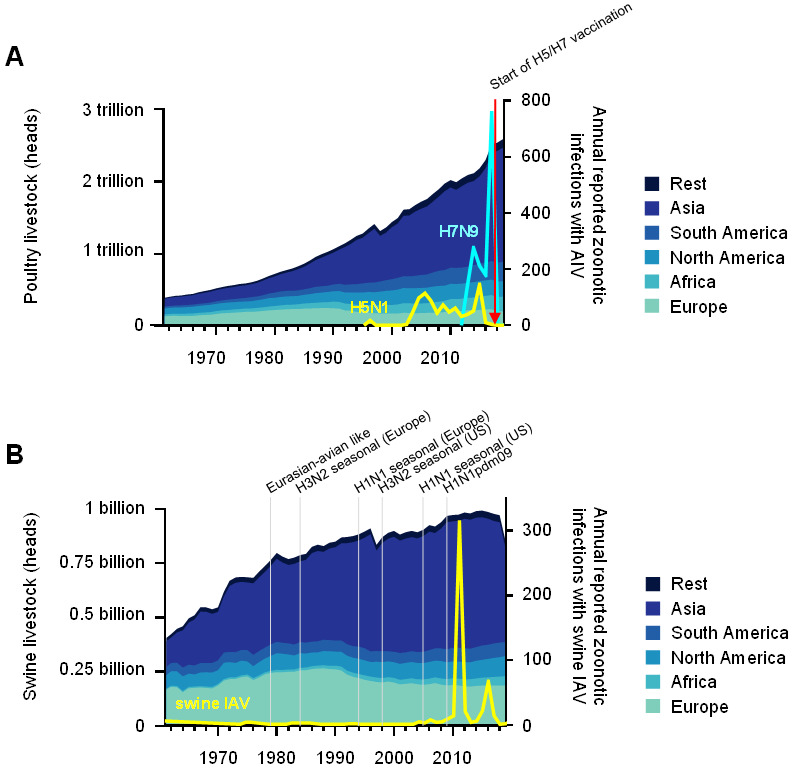
Poultry and swine livestock increased worldwide since 1961, as well as zoonotic transmission events and the establishment of IAV lineages. (**A**) The amount of poultry that were produced per year is shown for different global regions as share of the worldwide production on the left *y*-axis. The right *y*-axis indicates the number of humans that were infected with AIVs (H5N1 cases in yellow, H7N9 cases in light blue). The red arrow indicates the start of the H5/H7 poultry vaccination campaign. (**B**) On the left *y*-axis, the amount of swine livestock in different global regions as share of the worldwide production per year is shown. The number of humans that were infected with swine IAV are shown on the right *y*-axis (yellow line). The establishment of major IAV lineages in swine is indicated. The data on livestock were retrieved from FAOSTAT.

The rapid growth of the human world population since the mid-20th century and increasing prosperity in many parts of the world had a massive impact on the expansion and intensification of livestock farming, especially on poultry production [61]. According to the Food and Agriculture Association (FAO) of the United Nations, global poultry livestock soared from 4 to 2600 billion animals that were produced per year between 1961 and 2019 (Figure 1A). The dramatic increase in poultry production was particularly dynamic in low and middle income countries, especially in Asia, and surpassed the production in high income countries in the late 1980s [62,63]. To date, more than 60% and 80%, respectively, of the global domestic duck and goose populations are being produced and reared in China [64]. At the same time, the increasing incidence of infectious diseases, including emerging HPAI, have been a cause for concern. From 1959 to 2018, at least 29 different HPAIVs have emerged in poultry farms. These HPAIV strains emerged on nine occasions between 1959 and 1990 (one outbreak every ~3.5 years), causing insignificant loss and rarely being transmitted beyond the index case. In contrast, 20 genetically distinguishable HPAIV strains have emerged in poultry in the 29-year period after 1990 (one outbreak every ~1.5 years), resulting in hundreds of millions of birds that died or were culled [65,66,67,68,69,70].

The emergence of an HP H5N1 virus in 1996, which was reported to have infected a flock of geese in the Chinese province Guangdong, started a new age of HPAI. This lineage, termed gs/GD, has continued to evolve and expand ever since and has been responsible for an unprecedented number of outbreaks and transcontinental spreading events excluding, until now, only Australia, South America, and Antarctica. In addition, some of the descendant gs/GD lineages revealed substantial zoonotic potential. In 1997, the first human cases of HP H5N1 virus infection were reported, following a previous AIV outbreak among poultry in Hong Kong. Intriguingly, the H5N1 virus that was isolated from a 3-year-old boy from Hong Kong exhibited a high degree of genetic similarity to an HP H5N1 virus that was isolated in the preceding outbreaks in poultry, suggesting direct spillover of the virus [71,72,73]. Direct transmission of HPAIVs from infected poultry to humans has been implicated with sporadic infection in humans in the subsequent years [74,75,76,77,78,79,80]. The experimental data from the groups of Fouchier and Kawaoka showed that in 2012, an avian-derived HP H5N1 could acquire the ability for airborne human–human transmission by only a few mutational changes [81,82], fueling the concerns that these viruses might become pandemic. During the 19-year period from 2003 to 2021, 862 human cases of the HP H5N1 infection, with a case fatality rate of 53%, were reported (Figure 1A), the majority of them occurring in Egypt, Vietnam, and China [14].

In early 2013, the first identified cases of human infection with a novel LPAIV of the H7N9 subtype occurred in the Yangtze Delta region [83,84,85]. Phylogenetic analyses suggested that this H7N9 virus might have originated in poultry and then circulated in live-poultry markets, where it was eventually transmitted to humans through direct contact [86]. As of 2021, a total of 1568 confirmed human H7N9 cases had been reported (Figure 1A), with a case fatality rate of 39% [13]. To date, H7N9 outbreaks remain confined to China. While most of the infected persons were either poultry workers or recently exposed to poultry [87], experimental data as well as in-field studies suggest that H7N9 has a limited ability for a non-sustained human–human transmission following prolonged and intimate direct contact [79,88,89]. Serological studies that were carried out between 2001 and 2016 revealed a relatively low seroprevalence for antibodies to H5 (<4%) and H7 (<0.9%) in poultry workers and villagers in China and Southeast Asia [90,91,92,93,94]. Importantly, the introduction of a bivalent H5/H7 vaccine for poultry in the second half of 2017 dramatically reduced both the H7 positive rates in poultry and the number of human H5N1 and H7N9 cases [95,96]. After its first emergence in 2010, HPAIV of the H5N8 subtype circulated in global poultry populations and caused outbreaks in poultry flocks from 2014 onwards, first in South Korea and Japan and then in Europe and the US [97]. In 2021, Russian authorities reported that seven poultry workers were infected with a HP H5N8 virus in an H5N8 outbreak at a poultry farm in southern Russia [98]. There has been no indication for human–human transmission as yet and the reported human infections proceeded asymptomatically. This virus and its closely related descendants caused the largest ever HPAI epizootic outbreak in wild birds and poultry in Europe in 2020/21, however, no further human cases were detected. In 2010, poultry abattoir workers were reported to be infected with an IAV of the H10 subtype, following an outbreak of LPAIV H10N7 in an Australian chicken farm [99]. Since then, three more cases that were caused by an H10N8 and H10N3 virus were documented in China [100,101,102]. Notably, AIVs of the H10 subtype are highly diverse and are regularly found in poultry, ducks, and in live bird-markets [103,104,105]. In addition, occasional spillover infections with LPAIVs of the H9N2 subtype leading to mild disease in humans have been reported since 1998 [106,107,108]. Interestingly, the prevalence for H9-specific antibodies among people with frequent contact to poultry was found to be higher (<9%) compared to the prevalence of H5 and H7 antibodies in the same group. H9 specific antibodies were also found to be present in the general population (<3%) [90,92,94]. Fortunately, none of the above-mentioned avian IAV-subtypes that have been transmitted to humans in the past were found to have reassorted its genomic information with human-adapted IAVs in the field to date.

## 5. Mammalian Reservoir Species

### 5.1. Horses

Horses are a reservoir for two mammalian-adapted IAV-lineages, the H7N7 (historic) and H3N8 subtypes (contemporary) constituting the equine-1 and equine-2 lineages, respectively. In 1956, an IAV strain of the H7N7 subtype belonging to the equine 1-lineage was isolated from a horse in Prague following an epizootic outbreak in former Czechoslovakia [109]. The virus spread through the US in the 1960s, causing only relatively mild diseases. In 1963, following an IAV outbreak in racehorses in Florida that affected 60–70% of the animals, a H3N8 virus was identified and designated as the equine 2-lineage [110,111]. However, molecular clock analysis revealed that the equine H7N7 lineage had already emerged in the middle of the 19th century, coinciding with the 1872–73 horse epizootic outbreak [112,113]. Similarly, a time-calibrated phylogeny of the H3N8 equine lineage genomic surface glycoprotein sequences revealed that they emerged during the 1800s [112]. As reviewed by Morens and Taubenberger, horses were probably a major reservoir for IAVs before the 20th century and transmission between horses and humans may have occurred frequently [114]. One major event, thought to be a form of influenza due to the displayed symptoms and disease progression, was the same as the aforementioned 1872–73 horse epizootic event, which spread through the US along the rail lines where horses were transported [115]. An extraordinarily high mortality and morbidity rate in horses paralyzed the country, as travel and transport by horses had to be stopped. During this time, cases of influenza in humans were often linked to exposure to horses [114]. As outlined above, a horse-derived H3N8 virus is thought to have caused the 1889–1890 human pandemic. Sero-archaeological studies that were carried out in 1965 found antibodies that were reactive to the equine H3N8 subtype in elderly people [24,25,26]; more than 80% of the tested people born before 1891 had specific antibodies. Importantly, this was before the first documented introduction of an H3 virus into the human or swine population in 1968, fueling the assumption that an H3 virus circulated around 1890. The last widespread influenza epizootic in horses was in 1915/16 [114,116]. Notably, the world horse population peaked between 1910 and 1920 with 110 million horses and declined to ~60 million to date [64,117]. In the US, the horse population peaked with ~25 million horses in 1914 and then declined until 1964 (~1.5 million horses) and finally increased again to about 10 million horses to date [118]. In contrast, the European horse population has declined consistently [64]. While the H7N7 strain probably became extinct in the 1970s [119], the H3N8 strain still evolves globally in horse populations. As yet, no infections of humans have been reported. However, several more recent studies have found antibodies against equine H3N8, mainly in people with exposure to horses. Nevertheless, the antibody titers were very low and a cross-reactivity to human seasonal H3N2 viruses cannot be excluded [120,121,122]. Experimental infection of volunteers in the 1960s showed a general susceptibility to equine IAVs with asymptomatic or mild disease progression in two thirds of the study participants [123,124,125]. In addition, the H3N8 equine viruses have gained access from equine to canine populations on at least two occasions in the United Kingdom in 2002 and in the US since 1999 [126,127,128]. While only limited dog–dog spread was reported from a few kennels in the UK, the US canine cases developed into regional epidemics with cases being detected until at least 2016 [129].

With the onset of industrialization and mechanization, horses were no longer required to assist in tasks such as transportation or agricultural work. Today, the overlap between humans and horses is limited to leisure activities and sport [117]. Despite being an important reservoir in pre-industrial times, in a globalized and highly modernized world the importance of equine species as IAV reservoir has therefore decreased because of a declining population. Other animals, such as swine, whose numbers have steadily increased, have gained in importance.

### 5.2. Swine

Today’s diversity of IAVs in swine is the result of multiple historic and on-going spillover events, mainly from humans to swine and the viruses’ subsequent genetic drift and shift [130]. To date, H1, H3, N1, and N2 subtypes have been regularly isolated from pigs of all continents [131], which is strikingly similar to the past and present endemic subtypes in the human population (H1, H2, H3, N1 and N2). The first report of influenza in swine was in the US during the 1918 pandemic where John Koen observed that pigs exhibited the same symptoms, such as fever and coughing, and had a similar course of disease as influenza diseased people. Furthermore, he noted that a family’s influenza outbreak was followed by the infection of their pigs and vice versa [132]. The introduction of the pandemic 1918 virus into American swine populations, which was first isolated in 1930 from pigs, led to the establishment of the first endemic swine IAV lineage designated as “classical-swine” H1N1 [133]. However, cross-species transmission has led to the introduction of additional H1 lineages in swine. The “Eurasian avian-like swine” H1 lineage was established as the result of a spillover of an avian-derived H1N1 virus that was circulating in Northern Europe in the late 1970s [134]. Furthermore, several “human seasonal” H1-lineages were established following spillovers of human seasonal H1 viruses into swine herds. Human seasonal H1N1 viruses were first recognized in the European swine populations in the 1990s [135]. Other seasonal H1 viruses were introduced into the US swine populations by two independent events and were first identified in 2005 [136,137]. Besides the three H1 lineages (“classical-swine”, “Eurasian avian-like swine”, and “human seasonal swine”), at least two H3N2 lineages circulate in swine. Following the emergence of human H3N2 in 1968, seasonal human H3N2 strains spilled over into swine populations on many occasions, but largely failed to establish sustained long-term transmission in pigs [138]. Nevertheless, since the mid-1980s, two swine H3N2 lineages have been established independently. First, in 1984 a reassortant of the human seasonal H3N2 and the “Eurasian avian-like swine” lineage emerged in European swine holdings. Second, in 1998, when a triple reassortant between human seasonal H3N2, the “classical-swine”, and an avian IAV strain emerged in the US [139,140]. Reassortment events between the swine H1N1 and H3N2 lineages occur regularly and give rise to viruses with distinct genotypes that co-circulate among swine [141]. It is interesting to note that, as the only exception, the human pandemic H2N2 virus apparently has not been transmitted into swine populations.

Strikingly, all of the swine IAV lineages, with the notable exception of the classical-swine H1N1, were established after the 1970s (Figure 1B). Since then, pork production has been restructured, with small farms continuously being replaced by large production systems and animals being kept in stocks with ever-increasing population sizes. Pigs are widely transported across Europe and the world, providing connectivity between different swine populations and facilitating the global distribution of swine IAV strains [142]. For instance, the introduction of swine H1N1 into Japanese pig farms could be linked to the import of breeding stocks from North America [143]. Moreover, phylogenetic analysis of swine IAVs from samples from the US swine populations revealed that the viral spread of swine IAVs followed the transportation lines from the south to the Midwest [144]. Similarly, the transmission pattern of the Eurasian avian-like swine H1 subtypes in Europe was suggested to be trade-related. Nevertheless, regional patterns of spread remain conserved within Europe [145]. In addition, there are almost no documented migration events of swine IAVs between the European and American continents. This is in stark contrast to Asia, where strains of both origins and reassortants between them co-circulate [10]. Little is known about the status of pigs in Africa [146].

Even though swine IAVs were widely distributed in global swine populations between 1958 and 2009, there were only few reports about zoonotic infections of humans. A total of 73 zoonotic swine IAV infections with a case fatality rate of about 10% were reported in that period [107,147]. Thereof, twelve symptomatic and one lethal case were reported in the military base Fort Dix in the US in 1976 [148,149]. All of the diseased persons were male and previously healthy. No previous exposure to swine was reported and it is unclear how the virus was introduced into Fort Dix. Remarkably, a retrospective serological study revealed that the virus had spread through the unit and up to 273 soldiers were infected with the swine-derived H1N1 virus of the “classical-swine” lineage at that time [150,151]. Aside from this, a total of 60 civilian cases of swine IAV in humans, mainly young patients, have been reported in North America, Europe, Russia, and Asia. Most of the cases were caused by different circulating or reassorted swine IAVs of the H1N1 subtype and only a few by H3N2 or H1N2. While the majority of these cases had direct contact to swine, some cases were of unknown origin and human–human transmission in small clusters was assumed as well [147].

In 2009, a swine derived H1N1 virus from Mexico led to the latest influenza pandemic. Interestingly, the precursor of the pandemic virus is thought to have originated from Central-West-Mexico, the region with the highest density of swine in the country [12]. Notably, the virus was the result of multiple reassortments and contained avian-, swine-, and human-derived gene segments [40], highlighting the potential of swine serving as “mixing vessel” for viruses of swine, human, and avian origin. It is suspected that the global swine trade has contributed to the viral diversity in Mexican swine, which gave rise to the multiple reassortment virus [12]. Following the 2009 pandemic, globally occurring reverse zoonotic events re-introduced the 2009 pandemic H1N1 (H1N1pdm) virus into swine populations giving rise to novel reassortants and an increased genetic diversity [152]. Since 2011, three surveillance studies have independently identified such reassortant viruses in Chinese and European pig farms [145,153,154]. In China, six distinct H1N1 genotypes were reported, whereas 38 distinct swine IAV genotypes with 15 new combinations of different HAs and NAs were found in European swine populations. Of note, most of these new variants possessed human-preadaptation and several isolates were antigenically distinct descendants of the seasonal H1N1 strain in humans [145,153]. These studies not only demonstrated the high abundance of IAVs in the swine population, but also highlighted that simultaneous infection with different IAV strains does occur, drives reassortment, and creates new virus variants which pose a threat to the human population. In 2011, reports about clustered zoonotic transmission events with a novel variant H3N2 (H3N2v) virus containing the M segment of H1N1pdm were described [155]. To date, 429 infections with H3N2v and additional occasional infection with swine H1N1 and H1N2 have been reported, increasing the total number of human infections with swine IAV to 533 [107,147,156]. This drastic increase of zoonotic swine IAV cases since the 2009 pandemic might be the result of increased awareness and surveillance, or alternatively, it could be due to the increased presence of human-transmissible reassortant strains after the reintroduction of H1N1pdm.

In conclusion, swine only became an important reservoir for IAV of human origin due to increasing pork production, increasing livestock herd sizes, and intensifying transboundary transport. Moreover, the diversity of swine IAVs is constantly multiplied by the introduction of new, human-derived viruses [130].

### 5.3. Minks

Minks gained inglorious fame as a virus reservoir in the wake of the corona pandemic. In late 2020, it became widely known that SARS-CoV-2 had spilled over from humans to minks in fur farms where the virus then mutated and was transmitted back to humans [157]. This highlights that the human–mink interface potentially allows for viral spillovers. However, although various IAV subtypes have been found in minks, it is currently unknown whether IAV can be directly transmitted from minks to humans. An IAV of the H10 subtype was isolated from minks in the 1980s and it is believed to be the result of a direct transmission of an avian virus [158,159]. More recent studies have revealed a wider detection of several avian-, swine-, and human-derived IAV subtypes, such as H9N2 [160,161], H3N2 [162], H1N2 [163], and H1N1 [164,165] in minks. Additionally, infections with HP H5N1 were reported [166]. As shown recently, the H1N1pdm virus was not only re-introduced into swine but was also introduced into farmed minks [164,165]. Sun and colleagues reported a 50% and 11% prevalence of H1N1pdm and human H3N2-specific antibodies, respectively, in nearly 2500 serum samples from 34 mink farms in the Chinese Shandong and Hebei provinces, where the majority of the fur farms are located. Interestingly, antibodies against the AIV subtypes H5N6 (3%), H7N9 (3%), and H9N2 (20–50%), which have been endemic in poultry in this area at this time, were also detected in these mink-derived sera [160,161,164]. As 32% of the investigated sera contained antibodies to both human and avian strains, concerns arose that the minks could, similar to swine, serve as a mixing vessel [164]. Several studies suggested that avian- or swine-derived IAV virus were introduced to the minks by feeding them with raw poultry or pork by-products [161,162,163]. Usually, minks are kept in small cages right next to each other in holdings with up to 10,000 animals [167], facilitating aerogenic viral spread. Even though no transmission of IAVs from mink to human have been reported yet, the high diversity of IAV subtypes and their co-circulation in minks could give rise to new reassortant viruses with zoonotic potential. It should be noted that the closely related semi-domesticated mustelid species “ferret” is also used currently as the best animal model of human influenza infection. Ferrets proved to be highly susceptible to all of the human strains, they transmit virus by aerosols, and they generate highly strain-specific antibodies upon experimental infection [168].

### 5.4. Bats

In recent years bats have attracted increased attention for harboring a plethora of different viruses and are moreover suspected to be the reservoir for several lethal zoonotic viruses, such as Ebolavirus [169], Nipahvirus [170], and various Coronaviruses [171]. Numerous studies have linked the spillover of these viruses to humans with the manmade environmental fragmentation and deforestation that forces the resident bat populations from their secluded rainforest habitats into peri-urbanized landscapes [172,173,174]. There is, therefore, cause for concern that other zoonotic viruses of bat origin might become a threat to humans because of these environmental changes.

For a long time, bats were not considered to be involved in the IAV ecology. However, in 2012 and 2013 genomic sequences belonging to two distinct IAV subtypes, subsequently designated as H17N10 and H18N11, were isolated from swab samples of bats from Central and South America [175,176]. Serological testing of several bat species revealed a high prevalence of antibodies to H17N11 (38%) and H18N11 (50%), suggesting a wide distribution of these bat-derived IAVs in the Americas (176). Unfortunately, as serological data for other non-bat species, including humans, that share an overlapping habitat with these bats are missing, it remains unclear whether H17N10 and H18N11 are able to cross the bat-species barrier. Experimental infections recently showed that H18N11 has a limited ability to replicate in other species than its original host *Artibeus* spp. [177,178,179].

After serological studies discovered the presence of H9 antibodies in bats from Ghana [180], an H9N2 subtype was isolated from the Old World bat species *Rousettus aegyptiacus* in 2019 [181]. Interestingly, while Egyptian fruit bats could be experimentally infected with the bat H9N2 virus, they were resistant to infection with an avian-derived H9N2 virus [182]. Again, it is unclear whether bat-borne H9N2 can be transmitted to other species, like farm animals or humans invading the habitats of infected bats.

Despite bats being the second largest order of mammals, only little is known about bats as hosts of IAV. Further research is needed to elucidate the importance of the human-bat interface, including data on the seroprevalence of antibodies to the bat IAVs in humans who have had contact to infected bat colonies or who live in close proximity to the colonies.

## 6. Conclusions

Intriguingly, although IAVs circulate in a variety of different animal species, livestock animals are becoming especially important as a reservoir for zoonotic IAV transmission. As outlined above, the increased demand for animal products has driven the intensification of livestock farming and led to the introduction, adaptation, spread, and often endemic entrenchment of IAVs in these farmed animals. Consequently, the reported number of zoonotic infections increased in parallel to the extension and intensification of livestock rearing. While it cannot be excluded that zoonotic transmissions might occur also at the wildlife-human interface, the available data point towards a subordinate role of these interfaces in recent transmission events. Nowadays especially poultry and swine are reservoirs of concern. Although it is easier for large and integrated livestock holdings, in comparison to small backyard farms, to be equipped with improved biosecurity or biocontainment measures, an exchange of pathogens with the environment can never be fully prevented [183,184]. The assumption that these large holdings are important to prevent the introduction and release of potentially zoonotic pathogens is challenged: (i) Particularly large and dense populations with genetically similar animals facilitate viral spread between the animals [185,186]; (ii) rolling circle reproduction, especially in large swine holdings, favors the establishment of endemic pathogen circulation through the regular introduction of new susceptible host individuals [6,186]; and (iii) management of large livestock flocks requires huge logistics such as transport of live animals over larger distances which favors (global) pathogen dispersal [10,12,142]. Furthermore, improper ventilation systems and waste disposal could lead to pathogen release, which is especially critical in areas with a high density of animal production facilities [183]. Such conditions might foster less adapted strains to establish new lineages and new variants to emerge by reassortment events. In particular, swine were shown to be a potent ‘mixing vessel’ for IAVs of different species origins (e.g., swine, avian) and strains that are introduced by humans (e.g., “classical-swine” and “human-seasonal” strains). Thus, a zoonotic event that results in sustained human–human transmission, such as the one that caused the 2009 pandemic, is not unlikely. Minks were found to harbor an even higher diversity of avian-, swine-, and human-derived viruses, potentially forming another highly potent mixing vessel. Overall, intensified livestock farming has created an important interface between humans and animals and made zoonotic events that bear the risk of emergence of a human-transmissible virus more likely [184].

According to the “One Health Approach” human and animal health and the integrity of ecosystems influence and contribute to each other [187]. Functional ecosystems and the prevention of infections of animals is pivotal to reduce the risk of zoonotic spillover. Vaccines can be a very efficient tool to control IAVs in livestock. The nationwide use of a bivalent H5/H7 vaccine in poultry in China since the end of 2017 grossly reduced the incidence of H7N9 and HP H5 IAV infections and, consecutively, reduced the rate of human infections of H7N9 and H5N1 to near zero [95,96]. Vaccines against equine IAV are available as well. However, it is unclear whether the extinction of the H7N7 subtype in horses is a result of vaccination campaigns, since the last isolation was in 1979 and the first vaccination trials were in 1983 [188]. Interestingly, antigenic drift of equine IAV seems to be lower than for other IAVs, delaying need for an adaptation of the vaccine strains by several years [189]. In contrast, swine IAV are faster evolving and it is challenging to adapt vaccines accordingly. Inactivated vaccines often do not induce efficient cross reactivity and thus, are only efficient for the particular matching strain. Live vaccines can be more efficient but harbor a certain risk of regaining virulence by mutation or reassortment [190]. Here, vaccine strategies involving bat IAV, which cannot reassort with classic IAVs, could be useful [191,192]. Even though there have been promising vaccine strategies developed, unpredictable viral evolution remains the biggest challenge. Additionally, testing and quarantining of animals before introducing them to a new flock or location should be an effective measure. It also needs to be examined how a re-structuring of livestock rearing that favors less animals per space and smaller or more fragmented holdings in general could counteract viral spread. However, it should not go unnoted that increasing demands for “green” or bio-organic production partially goes along with an inherent conflict. In particular, poultry free range farming that interfaces to wild bird populations are widely opened and unfolded. Thus, risks for pathogen incursions from wild bird reservoirs are multiplied.

Nonetheless, the per capita consumption of pork, poultry, and eggs has constantly risen over recent decades [64,193] and is not likely to stop soon. To meet the needs of a growing world population with our limited resources and to manage the emergence of zoonotic diseases efficiently, we should rethink our consumption behavior regarding animal products and their production in general [194].

## Abbreviations

The following abbreviations are used in this manuscript:

IAV	Influenza A Virus
AIV	Avian Influenza A Virus
HA	Hemagglutinin
NA	Neuraminidase
HPAIV	High Pathogenicity Avian Influenza A Virus
LPAIV	Low Pathogenicity Avian Influenza A Virus
